# A large-scale study of peptide features defining immunogenicity of cancer neo-epitopes

**DOI:** 10.1093/narcan/zcae002

**Published:** 2024-01-29

**Authors:** Yat-tsai Richie Wan, Zeynep Koşaloğlu‐Yalçın, Bjoern Peters, Morten Nielsen

**Affiliations:** Department of Health Technology, Technical University of Denmark, Kgs. Lyngby, DK 28002, Denmark; Center for Infectious Disease and Vaccine Research, La Jolla Institute of Immunology, La Jolla, CA 92037, USA; Center for Infectious Disease and Vaccine Research, La Jolla Institute of Immunology, La Jolla, CA 92037, USA; Department of Health Technology, Technical University of Denmark, Kgs. Lyngby, DK 28002, Denmark

## Abstract

Accurate prediction of immunogenicity for neo-epitopes arising from a cancer associated mutation is a crucial step in many bioinformatics pipelines that predict outcome of checkpoint blockade treatments or that aim to design personalised cancer immunotherapies and vaccines. In this study, we performed a comprehensive analysis of peptide features relevant for prediction of immunogenicity using the Cancer Epitope Database and Analysis Resource (CEDAR), a curated database of cancer epitopes with experimentally validated immunogenicity annotations from peer-reviewed publications. The developed model, ICERFIRE (ICore-based Ensemble Random Forest for neo-epitope Immunogenicity pREdiction), extracts the predicted ICORE from the full neo-epitope as input, i.e. the nested peptide with the highest predicted major histocompatibility complex (MHC) binding potential combined with its predicted likelihood of antigen presentation (%Rank). Key additional features integrated into the model include assessment of the BLOSUM mutation score of the neo-epitope, and antigen expression levels of the wild-type counterpart which is often reflecting a neo-epitope's abundance. We demonstrate improved and robust performance of ICERFIRE over existing immunogenicity and epitope prediction models, both in cross-validation and on external validation datasets.

## Graphical Abstract

**Graphical Abstract:**
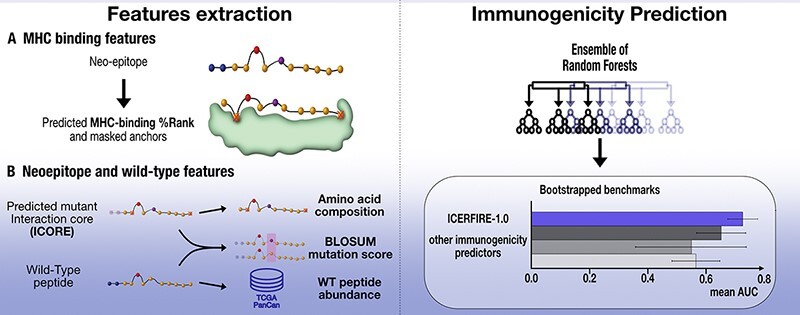

## Introduction

During tumorigenesis, malignant cells accumulate mutations which in turn may produce altered, tumour-specific proteins. Neo-epitopes are characterised by cancer-specific somatic point mutations in differentiated tissues and differ from other tumour associated antigens such as testis antigens, encoded by germline genes (1,2). Via antigen processing, peptides containing these mutations are generated, and if presented on major histocompatibility complex (MHC) molecules on a cell's surface, the resulting so-called neo-epitopes can drive antitumour T cell responses. One key component of this response is the CD8+ T cell-mediated killing of cancer cells displaying neoantigens on MHC class I (MHC-I). As such, T-cell therapy and cancer vaccines have emerged as promising approaches to treat malignancies by inducing tumour-specific immune responses (3), and ongoing efforts (4–9) have demonstrated the clinical efficacy of therapies targeting neo-epitopes. Hilf et al. developed neo-epitope vaccines for patients with glioblastoma, with personalised targeting based on mass spectrometry analyses of the immunopeptidome of the tumours, initiating both CD4+ and CD8+ T cell responses to mutated MHC-I neo-epitopes (5). Another study by Cafri *et al.* used tumour-infiltrating lymphocytes to identify neo-epitopes to construct mRNA vaccines for patients with metastatic gastrointestinal cancer, observing T cell reactions against some selected neo-epitopes in three of four patients tested (6). Whole-exome sequencing of tumour and normal cell DNA can also be used to identify neo-epitope targets (4,7–10). These approaches are highly cost intensive, as they rely on subsequent T cell reactivity screenings *in vitro* to validate the immunogenicity of the identified neo-epitope targets. Thus, there is a growing interest in predicting neo-epitopes using computational methods.

Neoantigens must go through a series of steps before they can be recognised by a T cell receptor on a cell's surface and induce an immune response. As a result, deciphering characteristics of neo-epitope immunogenicity requires taking into account a number of parameters. Firstly, a peptide's abundance is directly linked to the expression of its source protein, and several studies (11–16) have described an improved epitope identification through the inclusion of source proteins’ expression/abundance. Specific protein abundance levels can be estimated from a patient's tumour sample, for example through RNAseq. Alternatively general expression levels may be retrieved from publicly available databases such as TCGA (17) or GTEX (18). Additionally, tools such as PepX (19) have been developed to estimate a peptide's abundance from various databases.

Secondly, in the MHC class I pathway, the source protein is processed by proteases into peptides, before being bound to an MHC molecule and forming a peptide-MHC (pMHC) complex. The pMHC complex is transported to the endoplasmic reticulum by the transporter associated with antigen processing to be then displayed on the cell surface (20,21). MHC-I binding has been extensively studied and modelled. Consequently, there are multiple highly accurate computational models available for the prediction of MHC-I binding and antigen presentation (11,12,14,22–24). Given the importance of MHC presentation for T cell recognition, most epitope immunogenicity prediction tools integrate predicted MHC binding into their framework.

Finally, once presented on a cell's surface, various factors related to the neopeptide might impact T cell recognition. Neo-epitopes arise from somatic alterations of self-proteins, and as such, rules that apply for viral peptides immunogenicity might not hold true. Properties specific to neoantigens must be defined to appropriately characterise interactions between neoantigens and CD8 + T cells. One feature, for example, as noted in previous studies, is the dissimilarity to self, or the wild-type (WT) peptide (25–27). Here, the authors hypothesise that due to central tolerance, neo-epitopes too similar to its WT counterpart might be non-immunogenic. Considering T cell cross-reactivity, similarity to immunogenic peptides originating from previous infections were found to be linked to neo-epitope immunogenicity (27,28). Other studies found predictive power in the relative MHC binding affinity between a neo-epitope and its non-mutated peptide, defined as the agretopicity, being the ratio between the mutant and the WT’s predicted MHC binding affinity (26,27,29). Other studies showed an enrichment in hydrophobic or aromatic residues as being key characteristics for immunogenicity prediction in neo-epitopes (25,30,31). Finally, several studies observed the importance of various positions within a peptide sequence for the prediction of immunogenicity (25,26,28–30), suggesting that amino acid substitution or enrichment at certain positions might affect neo-epitope immunogenicity. For instance, Schmidt *et al.* developed PRIME, a immunogenicity prediction model that uses as input an amino acid frequency vector, computed from masking MHC-anchor position from an input peptide as well as the predicted MHC binding percentile rank, observing an enrichment in tryptophan in immunogenic peptides (30,32).

In this work, we performed a comprehensive investigation of this long list of proposed properties and features for prediction of cancer neoepitope immunogenicity using a neo-epitope dataset extracted from The Cancer Epitope Database and Analysis Resource (CEDAR) (33). We demonstrate improved prediction power by considering the optimal ICORE using NetMHCpan 4.1 (23) rather than the full peptide sequence. Optimal performance was found using the amino acids composition rather than the actual peptide sequence as input, and we show that weighting schemes such as masking or weighting MHC-anchor positions prior to computing amino acid frequencies to a high degree are dataset-specific, and that our model with an updated anchor masking method is able to better generalise and yields more accurate predictions across different datasets. Finally, we explored novel features computed using the ICORE, as well as previously defined features, identifying an optimal feature set and observed improved immunogenicity predictions, outperforming current methods for immunogenicity prediction or epitope prediction on three benchmark datasets.

## Materials and methods

### Training data

#### CEDAR mutant dataset

The raw dataset of cancer neo-epitopes was downloaded from the Cancer Epitope Database and Analysis Resource (CEDAR) in June 2022. Only HLA class I restricted peptides with full HLA resolution and annotated wild type were kept. We also excluded neo-epitopes resulting from frameshift mutations as our features based on mutation scores are computed for point mutations where mutant and wild type are aligned. We further filtered the mutants by allowing up to three mutations between the predicted MHC-binding ICORE and the aligned wild-type icore. This produced 3033 unique neo-epitope-HLA pairs, with 63 unique HLA alleles, of which 631 are positives and 2402 are negatives. As a peptide may come from different patients and bound to different HLA alleles, the dataset contains 2926 unique peptide sequences, restricted to one or more alleles.

#### Expression datasets

To further supplement our dataset, a second dataset was created by using the wild-type peptide's expression data. Expression data was obtained in January 2023, from the webserver of PepX, developed by the Immune Epitope Database (IEDB). The gene-level expression was collected using the wild-type peptides of each data point with PepX, and the TCGA Pan-Cancer Atlas as reference. The TCGA PanCanAtlas was developed using data generated by the TCGA Research Network available at https://www.cancer.gov/tcga. For the CEDAR dataset, in total, 107 data points (44 unique wild types) were removed as no expression data could be retrieved, leaving 2988 unique neo-epitope-HLA allele pairs, or 2882 unique peptide sequences. The expression of a given peptide was quantified and aggregated using three different methods: total gene TPM, total peptide TPM or total scaled peptide TPM as described by Frentzen et al (19).

### External data

Neo-epitope datasets were used as external evaluation datasets. All data were processed and filtered with the same method as the training dataset, keeping only linear peptides restricted to any of the 63 HLA class I alleles present in the training dataset, with full HLA resolution, as well as wild-type annotation, for lengths 8–12. Additional information specific to each dataset can be found below.

#### PRIME

The data used to train the PRIME method were downloaded from the supplementary materials of Schmidt *et al.* (30) in July 2022. To keep only neo-epitopes, the pathogen, random, and cancer testis peptides were discarded. To avoid overlap with the training data, data points that are present in both the training and the PRIME dataset were further removed. The filtered dataset consists of 2706 peptides (2664 negatives, 42 positives) for the base dataset, and 2591 peptides (2550 negatives, 41 positives) for the expression dataset.

#### NEPDB

Neo-epitopes were downloaded from the neo-epitopes database (NepDB) (34) on January 19th 2023 and used as a second external validation dataset. To remove any overlap with the training data, the peptides common to both the training and evaluation datasets were removed from the evaluation dataset and duplicates were removed. The filtered dataset consists of 243 peptides (219 negatives, 24 positives) for the base datasets, and 232 peptides (213 negatives, 19 positives) for the expression dataset)

### Training data partitioning

The training datasets were partitioned into 10 folds and used for nested 10-fold cross-validation. To avoid data leakage between partitions, the redundant data points were identified using the Hobohm-1 algorithm (35) and initially set aside. The similarity between two peptides was computed using the peptide kernel similarity algorithm (26,36) with window sizes of 3–8, and a threshold of 0.9 was set to filter similar peptides. The resulting dataset of dissimilar peptides were randomly split into 10 partitions. Finally, the held-out pool of similar peptides were iteratively added back to the corresponding by first adding back the identical peptides to the fold of their duplicate, then reassigning the related peptide to their match, by taking the maximum similarity computed above.

### Data processing

The MHC-binding ICORE of each mutant was predicted using NetMHCpan-4.1 with a pseudo-fasta format where the full peptide is treated as a protein sequence. This was done with a window length of 8–12 to find the optimal ICORE. The best ICORE was then picked by keeping the ICORE with the best predicted (lowest) percentile rank for its given HLA allele. Using the starting position of the mutant ICORE, the aligned wild-type ICORE was found, and used as the reference wild-type sequence from which features can be computed.

The position specific weights were assigned from the information content calculated from a set of natural human peptides, filtering for strong binders at a %Rank threshold of 0.25 to a given HLA predicted by NetMHCpan-4.1. From this set of predicted binders, position specific amino acid frequencies were calculated using Seq2Logo (37) excluding sequence weighting and applying a low count correction of 20. Next, the position-specific Kullback-Leibler (KL) information content was computed of each allele and lengths.

In total, five groups of weighting schemes were used prior to computing the amino acid frequency vectors. To prioritise non-anchor positions, ***Normal*** uses (1 – IC) as a weight vector and ***NormalMask*** uses (1 – IC) and a threshold of IC ≥0.2 to determine anchor positions to mask. In order to favour anchor positions instead, ***Inverted*** uses the IC as a weight vector, and ***InvertedMask*** uses a threshold of IC <0.2 to determine the non-anchor positions to mask. Finally, ***None*** applies no weighting to the sequence.

### Feature processing

To obtain the amino acid frequency vector, sequences are encoded using either OneHot encoding or BLOSUM62 encoding, and weights are applied by multiplying encoded sequence and weight vector, using any of the five weighting schemes. Depending on whether the full peptide or the ICORE was used as input, their corresponding full peptide %Rank or ICORE %Rank were added as features. The resulting vector of dimension 21 is used as the base input for our machine learning models. When using the peptide expression models, combinations of either of the three expression levels (total gene TPM, total peptide TPM or total scaled peptide TPM) were also added as features.

### The scaled agretopicity was computed as


(1) \begin{equation*}Agretopicity\ = \ \left| {Ran{k}_{Mut\ } - \ Ran{k}_{WT}} \right|\ \cdot \ \frac{{Ran{k}_{Mut}}}{{Ran{k}_{WT}}}\end{equation*}

We added this scaling factor to penalise instances where either the mutant or the wild-type peptide had very high ranks, i.e. they are non-binders, to prevent them from having the same ratio. For example, a mutant and wild-type with ranks of 0.1 and 1 respectively should not be treated the same as a mutant and wild-type who have ranks of 10 and 100 respectively, though they both yield an unscaled ratio of 0.1. This Rank-agretopicity, as the original affinity based agretopicity score, is expected to be low for immunogenic peptides, i.e. peptides where the rank score of the mutant peptide is low compared to the wildtype.

*Mutation scores* and *BLOSUM62 mutation scores* were computed using either the codon mutation matrix or the BLOSUM62 LogOdds matrix. Sequences were aligned using either the full peptide against its wild type, or the predicted ICORE and its aligned wild-type ICORE. The score of mutations within the aligned sequences were computed using the tables and summed.

The similarity score between a peptide and its wild type were computed using the peptide kernel similarity algorithm as described in Bjerregaard *et al.* (26,36), with K-mer windows of lengths 3–8 for both the full peptide and the ICORE.

Physico-Chemical properties for either the peptide or ICORE were computed using the *peptides* packages on Python 3.10. The following four features were selected based on the properties described. The features describe the aliphatic index, hydrophobicity, isoelectric point and the Boman potential protein interaction index (38–41). The features were chosen after analysis with feature correlations and one-sided Mann–Whitney and Welch tests.

The foreignness score as described by Wells *et al.* (27) was computed using a Docker installation of their *antigen.garnish* pipeline (version 2.3.1), installed and used according to the authors’ instructions at https://github.com/andrewrech/antigen.garnish.

### Human proteome data

The human proteome was downloaded from UniProt. Each protein was split into peptides of lengths 8–12 and their eluted ligand %Rank were predicted for each of the 65 HLA alleles present in the dataset (see Supplementary Table S1) using NetMHCpan 4.1. Random human peptides were used as background predictions for our model. Datapoints were sampled from the human proteome dataset, with the number of peptides sampled assigned to each allele and length according to their distribution in the original CEDAR dataset. Expression values (total gene TPM) for each of these human peptide samples were retrieved from the TCGA-PanCan (17) database using PepX (19). Data points containing missing expression values were omitted, resulting in 108 372 unique data points. Subsequently, single positions were randomly mutated to generate mutant peptides. Finally, the same feature processing methodology described above was applied to these random peptides, extracting the mutant ICOREs, aligned wild-type ICOREs and their associated features.

### Models and training

A nested 10-fold cross-validation scheme was used to train Random Forest models. This produces an ensemble of 90 models, which are then used for ensemble predictions. Random Forest models were developed using Python 3.10 and Scikit-Learn 1.1.1. Hyperparameters were tuned using the base features of unweighted amino acid frequencies and percentile rank in a 8-fold standard cross-validation. The final Random Forest model used 300 estimators, a max depth of 8, minimum of 7 samples per leaf, and a regularising factor alpha of 1e^−5^. An extensive grid of input encoding, weighting scheme and features were tested, and the optimal models CEDAR and PRIME models were selected based on the models’ performance. To allow statistical analysis of performances during the data processing and feature selection process, the cross-validation performance of each condition was taken and bootstrapped for 10 000 rounds. Then, a condition C1 was considered to perform significantly better than a condition C2 by doing a bootstrapped *t*-test on the AUC values for each round, where the *P*-value equals the 1 – (number of times C1 outperformed C2), divided by the number of rounds, i.e.


(2) \begin{equation*}p = \sum\nolimits_{n = 0}^{10000} {\frac{AUC_{c1} >AUC_{c2}}{10000}} \end{equation*}

### NNalign

As a baseline method and in an attempt to identify motifs from immunogenic peptides, NNAlign 2.1 was trained on the CEDAR dataset. The model used a motif length of 8, 50 hidden neurons, and trained for 75 epochs. The dataset was split by ‘common motif’ using NNAlign's setting and used a 10-fold nested cross-validation, with a burn-in period of 100 and early stopping. Data was encoded using both OneHot and BLOSUM encoding, and peptide lengths were encoded with individual neurons for lengths 8–12.

For the evaluation of difference in performance between approaches relying on amino acid composition and those rooted in sequence motif-based techniques, we additionally trained NNAlign models using varying motif lengths from 5 to 9, 50 hidden neurons, trained for 75 epochs in a 10-fold nested cross-validation. These models were trained using either the complete peptide or the ICORE as input. A prediction score was computed using a weighted average between the NNAlign score and (1 – %Rank) value from NetMHCpan 4.1. In this evaluation, we systematically tested weights spanning the range from 0 to 1, with increments of 0.05.

### Predictions benchmark

Various bioinformatic tools were used to benchmark our method on the training set (CEDAR) (33), external test sets from PRIME (32) and NEPdb (34). We first looked at MHC-binding predictors, which were used to assess the performance gain beyond MHC presentation alone, namely NetMHCpanEL 4.1 (23), MHCflurry 2.0.6 (24), MixMHCpred 2.2 (32) and HLAthena (without expression, referred to as HLAthena) (11) were used to obtain percentile rank (%Rank) predictions. Then, predictors related to immunogenicity and epitope prediction were used, with PRIME 2.0 (32) and Calis *et al.*’s immunogenicity predictor (42). Finally, other methods for epitope prediction that use peptide expression data were also benchmarked, with AXEL-F (13) and HLAthena (with expression, referred to as HLAthena_E). In line with our analysis, each of these predictions were bootstrapped 10 000 times. The benchmark datasets and their respective prediction scores can be found in the supplementary data (See Table on next page).

**Table:** 

Software	Author/reference	Version	Misc.
NetMHCpanEL	Reynisson *et al.* (23)	4.1	Executed on Computerome-2.0
NNAlign_MA	Alvarez *et al.* (43)	2.0	Executed on Computerome-2.0
MixMHCpred	Gfeller *et al.* (32)	2.2	https://github.com/GfellerLab/MixMHCpred
PRIME	Gfeller *et al.* (32)	2.0	https://github.com/GfellerLab/PRIME
MHCflurry	O’Donnell *et al.* (24)	2.0.6	https://github.com/openvax/mhcflurry/tree/v2.0.6
HLAthena	Sarkizova *et al.* (11)	−	Executed locally with a Docker image http://hlathena.tools/
IEDB Calis	Calis *et al.* (42)	−	http://tools.iedb.org/immunogenicity/
AXEL-F	Koşaloğlu-Yalçın *et al.* (13)	1.1.0	Executed locally with a Docker image http://tools.iedb.org/axelf/download
NetMHCpanExp	Garcia-Alvarez *et al.* (12)	1.0	https://services.healthtech.dtu.dk/services/NetMHCpanExp-1.0/
AntigenGarnish	Richman *et al.* (25,27)	2.3.1	Executed locally with a Docker image https://github.com/andrewrech/antigen.garnish

## Results

**Figure 1.**
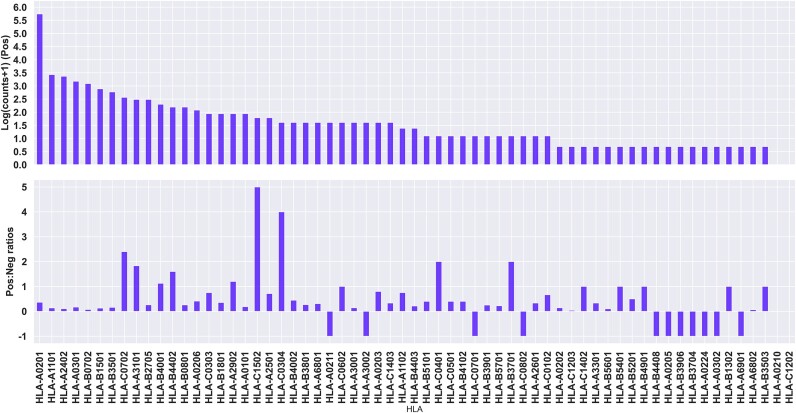
Quantitative description of the CEDAR data set. Top panel: Log positive counts, with an offset of +1 to account for alleles with 0 positive samples. Bottom panel: Positive to negative ratios for each allele. A ratio of –1 indicates no negative samples.

With the aim of developing machine learning models for the prediction of cancer neo-epitopes, we have collected data from **CEDAR** (33), focusing on HLA class I-restricted neo-epitopes. As the peptide properties (i.e. feature space) we want to explore relies on information from the wild-type (WT) peptide, we further restricted the dataset to keep only peptides for which the WT peptide annotation were available (for details refer to Materials and methods). The data set consists of peptides of length 9–12 restricted that include reported restrictions to 63 different HLAs. Out of those, the majority of the peptides (80%) are reported to be restricted to 7 HLAs (see figure 1A). This data set consists of 3033 unique peptide-HLA pairs, with 2926 unique peptides, as a given peptide can be bound by different HLAs, with 2402 (79%) negative and 631 (21%) positive data points (see Figure 1, Table S1).

### Impact of predicted HLA antigen presentation

**Figure 2.**
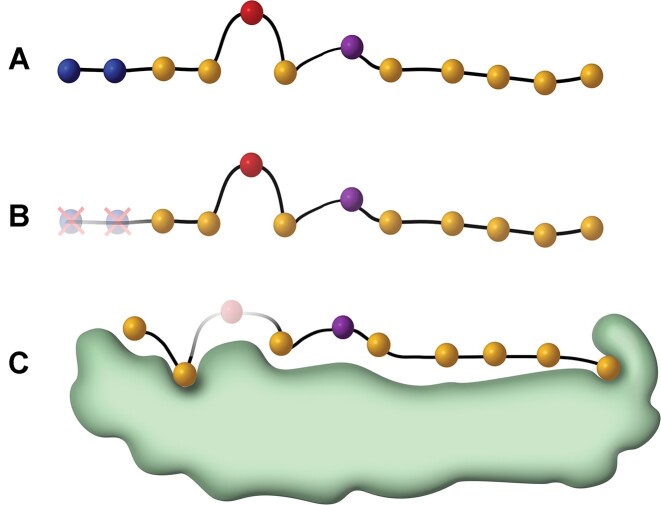
Summary of the input types tested. The ICORE is computed from the full peptide through NetMHCpan-4.0 and may include deletions at the N or C termini, as illustrated here. Panel **A** shows the full peptide prior to MHC binding and antigen processing. Panel **B** shows a submer after the predicted cleaving of N-terminal amino acids for MHC-binding, termed the ICORE. Panel **C** shows the MHC-bound submer, with the transparent amino acid in red being removed when only considering the CORE.

As epitopes require presentation on MHC to elicit an immune response from CD8 T cells, we first examined the performance of predicted MHC-binding for discriminating between positive and negative data using NetMHCpan-4.1 (23). We considered (i) a predicted %Rank using the full peptide (Figure 2A), and (ii) a sliding window within the peptide to find the optimal interaction core by picking the best scoring submer in terms of %Rank (ICORE, Figure 2B). Note that the ICORE is different from the CORE, in that the ICORE is the sequence of the peptide predicted to be bound and presented by the MHC, and the CORE the minimal 9 amino acid binding core directly in contact with the MHC (see Figure 2C). This analysis covered 854 instances where the predicted ICORE differed from the full length peptide and showed that the ICORE had a slight but significantly improved predictive power compared to using the full-length peptide (Supplementary Figure S1, ROC AUC = 0.634 and 0.611 respectively, *P* = 0.0007 using a one-sided bootstrapped *t*-test). This suggests that focusing on minimal optimal epitopes is advantageous even in author reported ‘minimal’ epitopes.

Since neo-epitopes are produced by cancer-specific mutations, we tested whether immunogenic neo-epitopes were enriched for either anchor or non-anchor mutations, and whether these mutations were located within the ICORE. To this end, we picked the best predicted-binding ICORE for each peptide together with the corresponding WT ICORE. To locate the anchor positions, a conservation score (i.e. information content, IC for each peptide position) was calculated from the position specific amino acid frequencies calculated from a large set of predicted binders for each HLA and peptide length from 8 to 12. Details regarding the generation of the information content weights can be found in the methods section. Next, an anchor position for a given HLA was defined as a position having an IC higher than 0.200. The threshold for defining anchor positions was manually chosen given the sequence logos and information content for 9-mers and are shown in Supplementary Table S2.

This analysis revealed that 116 peptides (<4%) had a mutation outside its ICORE, out of which only 11 (9.5%) peptides were found to be immunogenic, against 620 (21.3%) for immunogenic peptides with ICORE mutations. This result demonstrates that the subset of peptides with mutations outside the predicted ICORE is significantly depleted in immunogenic peptides (*P* = 0.0011, one-sided proportions z-test). Out of the 2917 remaining peptides with mutations within the ICORE, 73.5% of the mutations were located at non-anchor positions. The proportions of non-anchor mutations were not found to differ significantly between positive and negative peptides, with 71.6% for immunogenic and 74.0% for non-immunogenic peptides respectively (*P* = 0.1151, one-sided proportions z-test). Finally, for the 773 peptides with anchor mutations, 77.2% of them are negative and 22.8% are positive.

### Integration of amino acid composition

Several approaches for representing the peptide sequences into a machine learning model were next investigated, all based on different variants of amino acid composition. Schmidt *et al.* (30) described masking anchor positions as a means to only include TCR facing residue when computing the amino acid composition. An alternative approach, that does not entirely mask out parts of the sequence, would be to up or down weight the HLA anchor positions, using weight defined for instance from the position specific IC values described above for each HLA and peptide length. Given the similar propensity for anchor and non-anchor mutation for both immunogenic and non-immunogenic peptides, both methods of weighting were tested.

We investigated the various weighting schemes using either of the two input formats to derive the amino acid composition, i.e. the full peptide and its full peptide rank, or the best predicted ICORE and its associated rank. The different resulting amino acid composition vectors as well as the %Rank value were used as input for a Random Forest model with the purpose of identifying the optimal amino acid composition representation (for detail on the Random Forest, refer to materials and methods). To assess the relative performance difference between methods based on the amino acid composition and sequence motif-based methods, we also trained a NNAlign model using either the full peptide or the predicted ICORE as input. We computed a weighted average score by combining the NNAlign score and the %Rank value from NetMHCpan (either of the peptide or ICORE), but found that this method underperformed the amino acid composition method without any positional weighting, with the best full peptide version scoring 0.66 AUC and the best ICORE version scoring 0.70 AUC (data not shown). Details pertaining to the cross validation and the weighted averaging methods can be found in the Materials and methods section.

**Figure 3.**
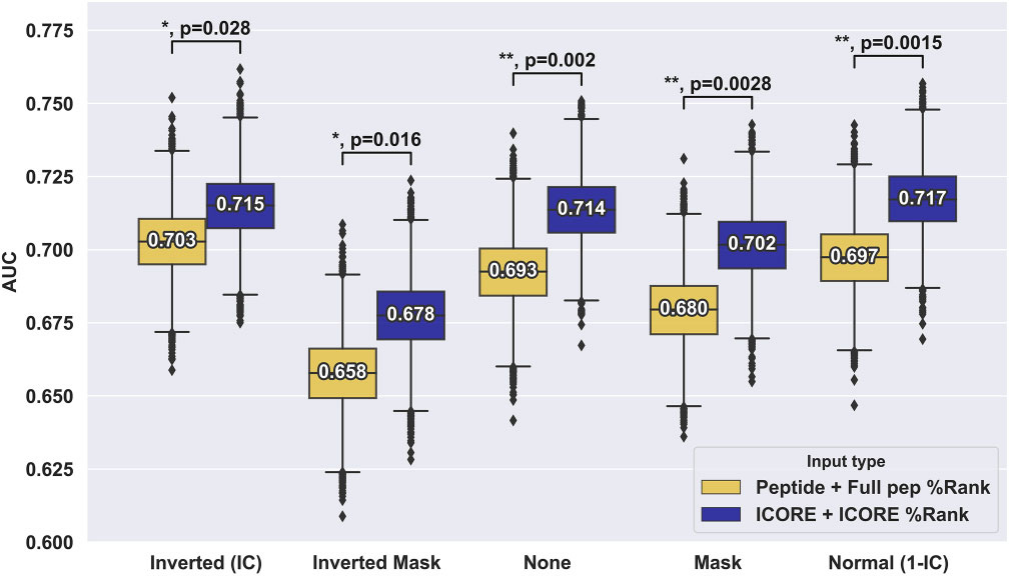
Performance evaluation of different positional weight schemes for the CEDAR data. For each of the weighting schemes, the %Rank and amino acid composition were derived from either the full length peptide or the ICORE, resulting in vectors of 21 features used in Random Forest models for cross-validation. Number labels indicate median values. Boxplot lines indicate 25, 50 and 75% quartiles respectively, whiskers indicate the 1.5 interquartile range for 10 000 rounds of bootstrapped cross-validation AUCS. Sig levels: * = 0.05, ** = 0.01, *** = 0.001, **** = 0.0001.

Several important conclusions can be drawn from these results. First and foremost, and in line with what was observed in figure S1, the models using ICORE and ICORE %Rank as input significantly outperformed the models using the full peptide and full peptide %Rank for all of the weighting schemes (see Figure 3). Second, the results demonstrate that the use of position masking in general performs suboptimal compared to the use of no weighting or IC-positional weighting. As expected, the Inverted Mask weighting scheme had the poorest performance, as all but the anchor positions are masked. In terms of positional weighting, the results show that all weighting schemes achieve comparable performance, with a slight, non-significant advantage in favour of anchor upweighting (KL weighting) (see Supplementary Figure S1). Additionally, other ways of computing the information content (KL or Shannon) were investigated and the same behaviour was observed for the two types (see Supplementary Figure S2).

The conclusions from the analyses in Figure 3 are opposite to what was proposed by Schmidt et al., where optimal performance was observed when anchor positions were masked out on a per-HLA, per-length basis. To investigate further if this discrepancy was imposed by our masking implementation or by the CEDAR data set used in the analysis, we investigated whether this effect held true when evaluating our models on the dataset from Schmidt *et al.* (30) filtered to only keep neo-epitopes.

**Figure 4.**
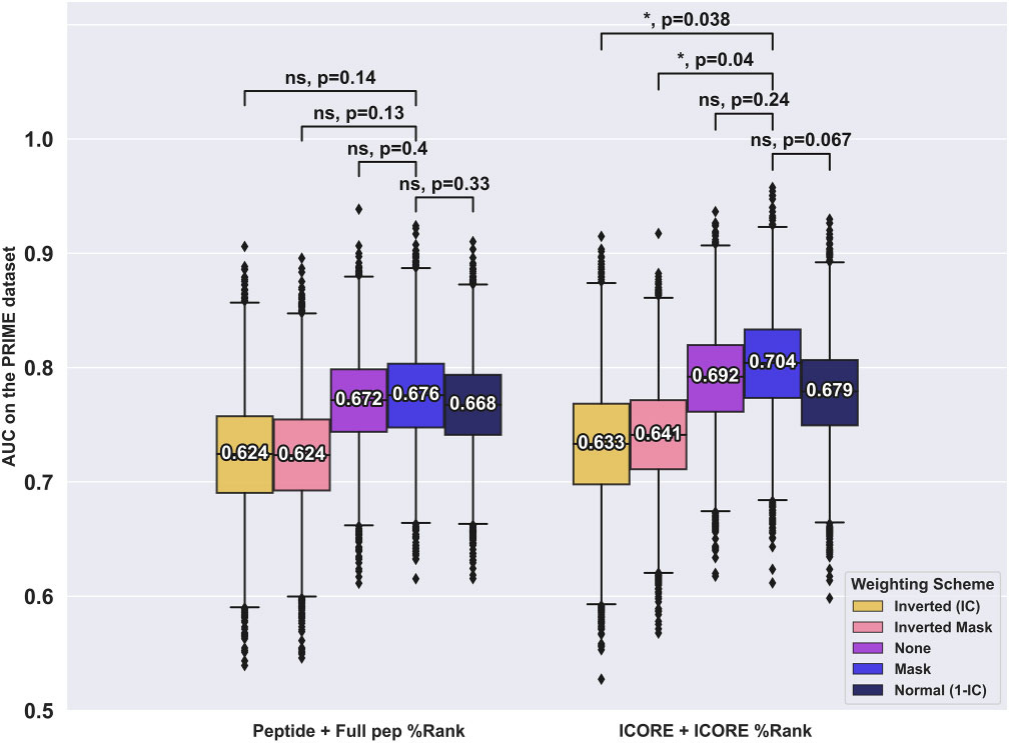
Performance evaluation of different positional weight schemes for the PRIME dataset. Each model was trained on the CEDAR dataset using either of the input and weighting methods as described above and evaluated on the PRIME dataset. Boxplot lines indicate 25, 50 and 75% quartiles respectively, whiskers indicate the 1.5 interquartile range for 10 000 rounds of bootstrapped cross-validation AUCS. Sig levels: * = 0.05, ** = 0.01, *** = 0.001, **** = 0.0001

For the PRIME data set, this analysis gave results in line with that of Schmidt et al. i.e. with the optimal method using anchor masking (see (see Figure 4). This result highlights a strong discordance in the weighting behaviour between the two datasets. This may be a result of inherent properties of the two datasets. One clear difference is the proportion of positive to negative peptides which is 0.26 for the CEDAR data and 0.04 for PRIME. However, in terms of anchor versus non-anchor mutations, the PRIME data set behaves very similarly to that of the CEDAR data, with 32% of both positive and negative peptides having anchor mutations.

Given this discrepancy and non-significant performance gain when using different weighting schemes, the model in the following analysis uses the simplest, unweighted input.

### Investigating additional features

To go beyond MHC binding, we next investigate the impact of other properties and features of neo-epitopes in our model. Earlier research has proposed that agretopicity (25–27,29), defined as the ratio between the binding affinity of a mutant and its WT peptide, as a feature associated with neo-epitope immunogenicity. Previous studies have underlined the improved performance and reliability of using %Rank scores rather than binding affinity values to characterise HLA-binding (44). Also, earlier work has demonstrated the improved predictive power of applying eluted ligand likelihood scores over affinities for the prediction of epitopes (45). Consequently, we used eluted ligand percentile rank scores rather than predicted binding affinity to construct a redefined rank-based agretopicity score (for details on this score refer to methods). Here, we used the ICORE predicted rank of the mutant peptide, and the predicted rank value for corresponding wildtype ICORE, and defined the scaled Rank-agretopicity score as the ratio of the mutant rank over the WT rank, scaled by the absolute value of the difference of the two (refer to materials). We also compared performance using the original definition of agretopicity based on the ratio between the binding affinity of the mutant and its wild-type counterpart and found that our rank-agretopicity outperformed the former (AUC = 0.589 and 0.539 for the rank and binding affinity ratios, respectively). As an alternative to this aggregated score, we further investigated if the binding potential of the WT peptide could add value to the model as a complement to the binding potential of the mutant variant. A summary of the distribution of mutant and wild-type ranks for both anchor and non-anchor mutation groups and labels in Figure S3.

Further, previous studies have suggested that both the similarity to known epitopes, termed the *foreignness score*, and similarity to self, computed by scoring peptides against the human proteome, share predictive power for the identification of neo-epitopes (25–27). Here, we used *antigen.garnish* (25) to compute the foreignness score. For self-similarity, we calculated the similarity between the mutant and wildtype ICORES, using a peptide kernel similarity defined by Shen et al (36) and as previously used by Bjerregaard et al (26). This similarity is computed by using sliding K-mer windows of lengths 3–8 along both sequences and using BLOSUM62 as a scoring matrix. This scoring scheme by construction gives less importance to either ends of the peptide, prioritising its centre. To characterise a given mutation, we moreover explored two different scores. The first is referred to as the *BLOSUM mutation score and* is defined from the BLOSUM score for each mutated amino acid between a mutant and wild-type sequence. The second is referred to as the *Codon mutation score* and is defined as the probability of a mutation as a result of a single nucleotide mutation, given the codon of the wild-type amino acid and the mutant amino acid.

Next, we looked at various physico-chemical properties of peptides. Earlier evidence has pointed at properties such as hydrophobicity having predictive power for immunogenicity (31,46). Another property related to hydrophobicity is the aliphatic index of a peptide, defined by Ikai et al. (41) as the relative volume occupied by aliphatic (and thus, hydrophobic) side chains within a peptide. Boman *et al.* described a potential protein interaction index (Boman index) (38), loosely related to hydrophobicity properties. Details pertaining to the extraction of physico-chemical properties can be found in the methods section.

Finally, we also included information related to source protein abundance of the peptides. As we do not have access to the raw sequence data for the neo-epitopes included in our CEDAR and PRIME data sets, we used the Peptide eXpression annotator (pepX) (19) to retrieve an estimate of expression level of the wild-type peptide for each mutant. Given the neo-epitope nature of our dataset, we limited the reference dataset to the TCGA Pan-Cancer database (17,47). Here, each dataset (CEDAR, PRIME) were further filtered to keep only data points for which expression value could be assigned (hereafter referred to as *X_expr* datasets). This filtering removed 45 data points from the CEDAR dataset (from 3033 to 2988 unique peptide–HLA pairs), and 156 data points from the PRIME dataset (from 2706 to 2550 unique peptide–HLA pairs). According to PepX recommendations, *Total Gene TPM* was applied in the subsequent analysis (19).

**Figure 5.**
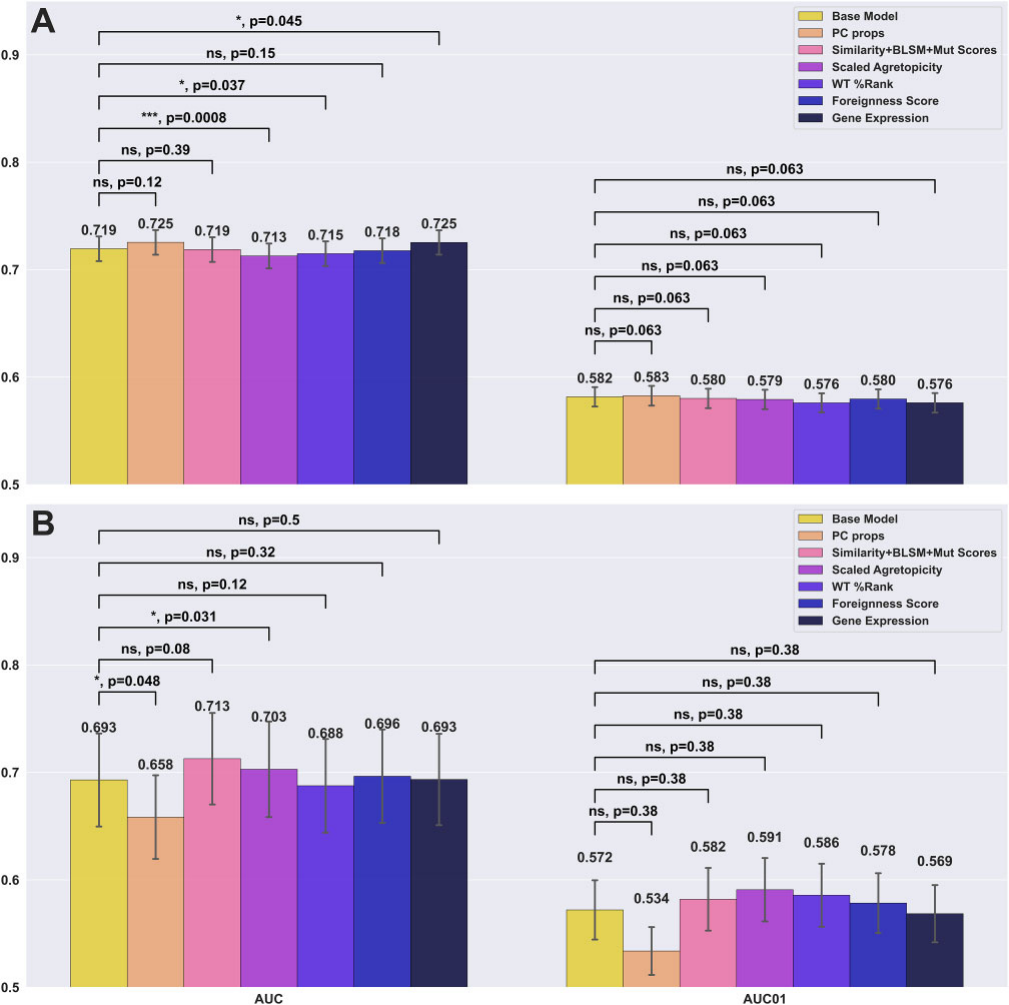
Performance Comparison of models integrating different additional features. Panel **A** reports the cross-validation performance on the CEDAR dataset. Panel **B** reports the external test performance on the PRIME dataset. Each bar indicates the addition of a given feature on top of the base 21 features (amino acid frequency + MUT %Rank) Each bracket indicates a bootstrapping *t*-test between the base model and an additional feature, where the null hypothesis is where the added feature does not improve performance compared to the base model.

A full grid search was performed to select the optimal combination of features. The distribution of each feature by immunogenicity and a correlation matrix of the features for the training dataset can be found in figure S3 and figure S4 respectively. Figure 5 summarised the results of this analysis using the best individual weighting scheme according to Figures 3 and 4 for each method, i.e. no positional weighting on the CEDAR and masking weighting for PRIME.

Here, the bootstrapped *t*-tests and their *P*-values compare whether a given additional feature improves performance over the base model, while the *P*-value for the opposite test, where the base model outperforms the model with an additional feature can be obtained by taking 1 minus the *P*-value of the first test. Examining the result in Figure 5, we first compared the performance difference between the baseline model (amino acid composition + %Rank) against each model expanded by additional features. When evaluated on CEDAR, only the models using physico-chemical properties and antigen expression as additional features outperformed the baseline model (ns, *P* = 0.1152, *, *P* = 0.0453, respectively), both reaching 0.725 in AUC compared to 0.719 for the baseline model. In contrast, when looking at performances of the models evaluated on the PRIME dataset, we noticed once again a divergent behaviour. Here, the model with added physico-chemical properties significantly underperformed compared to the baseline model (*, *P* = 1 – 0.9524 = 0.0476), dropping from 0.693 to 0.658 AUC) while the model with mutation-related features (self-similarity, BLOSUM mutation score, codon mutation score) improved performance with a gain of 0.02, though the gain was statistically insignificant (*P* = 0.08). Furthermore, the model using scaled agretopicity significantly underperformed the base model on the CEDAR dataset (***, *P* = 1 – 0.9992 = 0.0008), but significantly outperformed the base model on the PRIME dataset (*, *P* = 0.0307).

Finally, no change in predictive performance was observed on the PRIME data when adding expression levels. When looking at the other features tested, they seem to have little impact on the CEDAR dataset, while having a larger variation from the baseline on the PRIME dataset. This could be attributed to a bias or difference in features distribution across classes between the two datasets, or an artefact of overfitting during cross-validation given the small data set sizes. Together, these results suggest that while each feature set and weighting scheme can improve performance, this gain is dataset-specific, due to intrinsic biases.

### Model generalisation

Despite individual features not bringing a significant boost in performance, we performed an extensive search for combinations of various additional features and weighting schemes and defined the optimal model for each dataset (optimal CEDAR model, optimal PRIME model) as the best performing model when evaluated on the respective datasets yielding a combination of optimal features and positional weighting scheme. Next, these optimal models were compared to the baseline model, defined from the amino acid composition computed without any positional weighting from the ICORE and the mutant %Rank. Further, to complete the analysis, we evaluated an NNAlign (48) model trained on CEDAR, as well as the PRIME-2.0 (30,32) model on both datasets to underline problems with overfitting and generalisation. The NNAlign method is aimed at identifying linear motifs in biological sequences (48), and we hypothesised that immunogenic neo-epitopes might share common characteristics and sequence motifs (for details on this model, refer to materials and methods).

**Figure 6.**
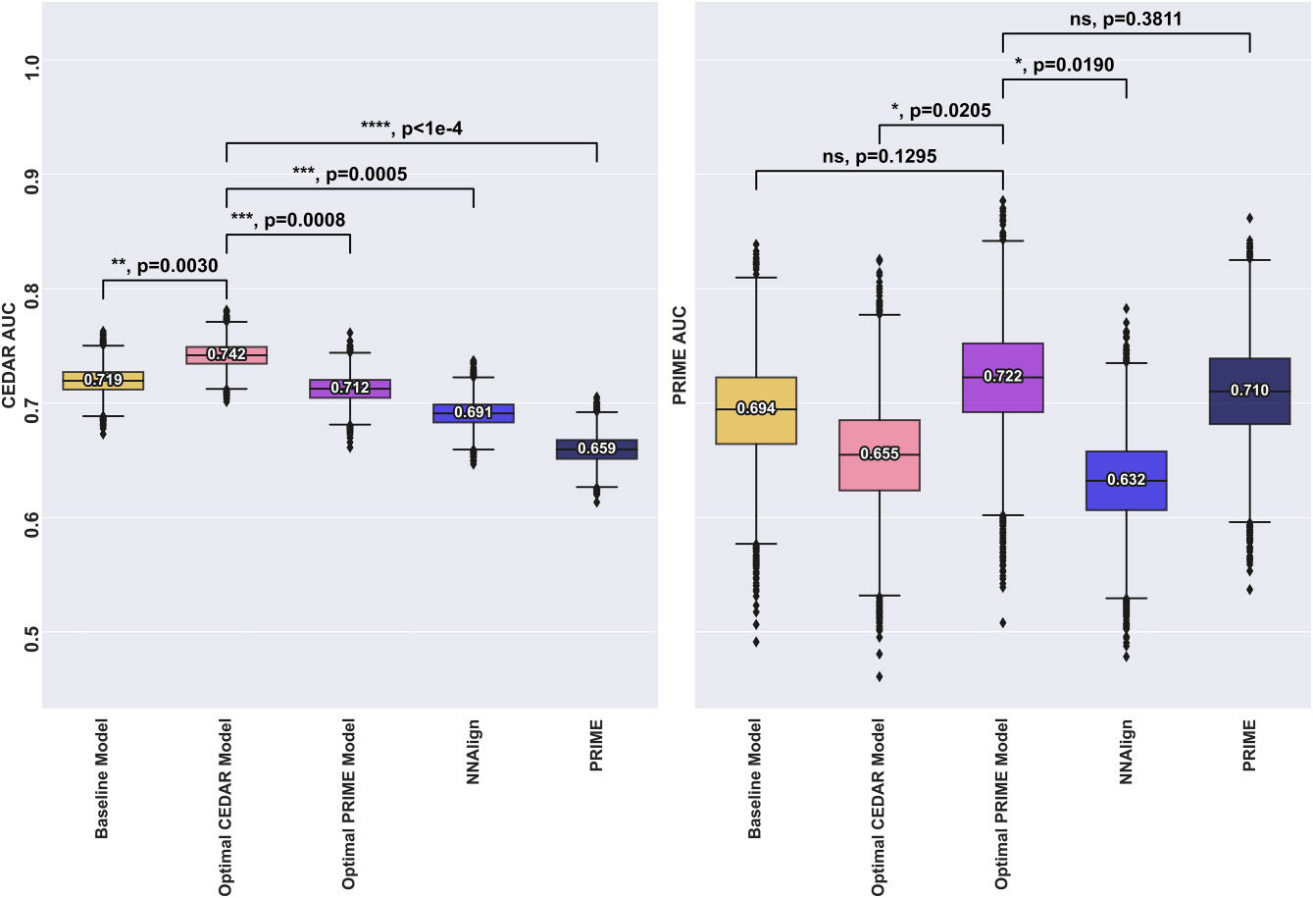
Comparison of the baseline model, optimal models for each dataset as well as NNalign and the PRIME-2.0 method for comparison. All models except PRIME were trained on the CEDAR dataset. Left panel indicates performance for models evaluated on CEDAR, right panel indicates performance for models evaluated on PRIME. Number labels indicate median value. Boxplot lines indicate 25, 50 and 75% quartiles respectively, whiskers indicate the 1.5 interquartile range for 10000 rounds of bootstrapped cross-validation AUCS. Black bars and *P*-values annotations compare the performance of an optimal model to all other models. Blue bars and p-values annotation compare the performance of the consensus model to other models. Sig levels: * = 0.05, ** = 0.01, *** = 0.001, **** = 0.0001

The result of this is shown in Figure 6. Here, the optimal CEDAR model was found to use ‘Inverted IC’ for positional weighting, upweighting the anchor positions, with the Boman Index, BLOSUM mutation score, the WT %Rank, foreignness score and antigen expression as additional features, outperforming the baseline model (**, *P* = 0.003). For the PRIME data set, the optimal model was found to use masking as positional weighting, with self-similarity, BLOSUM mutation score and scaled agretopicity and antigen expression as additional features, outperforming the baseline model (ns, *P* = 0.13). This result again underlines the fundamental difference between the CEDAR and PRIME data sets.

Although the optimal CEDAR model significantly outperformed all the other models on the *CEDAR* dataset, it underperformed all the other models when evaluated on the *PRIME* dataset except NNAlign. In particular, when looking at the performance of the optimal CEDAR model across the two datasets, the performance dropped from 0.742 to 0.655 AUC when evaluated on the *PRIME* dataset, significantly worse than the other models, suggesting a poor generalisation ability. This could be attributed to the unconventional positional weighting preferred by the model as well as the features it uses. In particular, the inclusion of Boman Index, WT %Rank and foreignness score as well as the ‘inverted’ weighting, placing importance on the anchor positions, only improved performance when evaluated on the *CEDAR* dataset. In contrast, the optimal PRIME model performed comparably on either datasets. Finally, as noted with the optimal CEDAR model, signs of overfitting can be seen with the other models. PRIME’s performance drops from 0.710 to 0.659 AUC when evaluated on the *CEDAR* dataset, suggesting that akin to our optimal CEDAR model, their model might be overfitting to an inherent bias of their training data. Similarly, we observe a performance drop for the NNAlign model trained on the *CEDAR* dataset, from 0.691 cross-validation AUC to 0.632 AUC when evaluated on the *PRIME* dataset. Together, these results show that models trained and selected on either dataset are likely to overfit to specific characteristics and fail to generalise.

### Evaluation of external independent data and consensus model

As the definition of an optimal model was found to differ depending on the dataset, we next attempted to define a model able to better generalise to any datasets. To do so, we first selected an external, independent dataset. We downloaded and filtered neo-epitope data from NEPdb (34) by excluding peptides included in the *CEDAR* training data set, selecting epitopes of lengths 8 to 12, restricted to MHC class I with wild-type annotations. For more details, refer to the methods section. To define an optimal, generalisable model, we trained models on CEDAR and picked the best model based on the harmonic mean between the cross-validation AUC on CEDAR and the test AUC on PRIME, yielding a ‘consensus model’. Prior to this, the overlap between *PRIME* and *NEPDB*, was removed from the *PRIME* dataset in order to prevent selecting a model that would have inflated performance on *NEPDB* during the benchmark. This filtering process yields a reduced *PRIME* dataset containing 2395 negatives and 36 positives, termed *PRIME reduced* in the following results. From this, we obtained the ‘consensus’ model, which was found to be similar to the optimal PRIME model, using ICORE as input, masking as positional weighting, BLOSUM mutation score and antigen expression as additional features. We term this consensus model, ICERFIRE, for ICore-based Ensemble Random Forest for neo-epitope Immunogenicity pREdiction. Similarly, an updated Optimal PRIME model was picked using the reduced PRIME dataset to avoid boosted performance on the NEPDB dataset due to the overlap between the two datasets, yielding a model that uses masking as positional weighting, self-similarity and BLOSUM mutation score as additional features. Furthermore, we explored the feasibility of training a model on a merged dataset of *CEDAR* and *PRIME reduced*. However, our investigation revealed that training on the combined dataset did not lead to enhanced performance (refer to Table S3). This behavior may be attributed to the imbalance in class distribution between *CEDAR* and *PRIME*. Consequently, the trained models tend to prioritize *CEDAR* data points due to its larger proportion of positives. Despite experimenting with various sampling strategies, such as up-sampling positives from the PRIME dataset or down-sampling *CEDAR* data points, the models did not exhibit improved performance.

We evaluated our models (ICERFIRE, Baseline Model, Optimal CEDAR, Optimal PRIME) as well as external methods, IEDB-Calis (42), PRIME (30), NNAlign (43) (trained on the CEDAR data, see earlier), NetMHCpan (23), MixMHCpred (32), MHCFlurry (24), HLAthena (11,14), AXEL-F (13) and NetMHCpanExp (12) and a foreignness score from *antigen.garnish* (25). All the methods were benchmarked on the data sets including estimated RNAseq expression values (see earlier).

**Figure 7.**
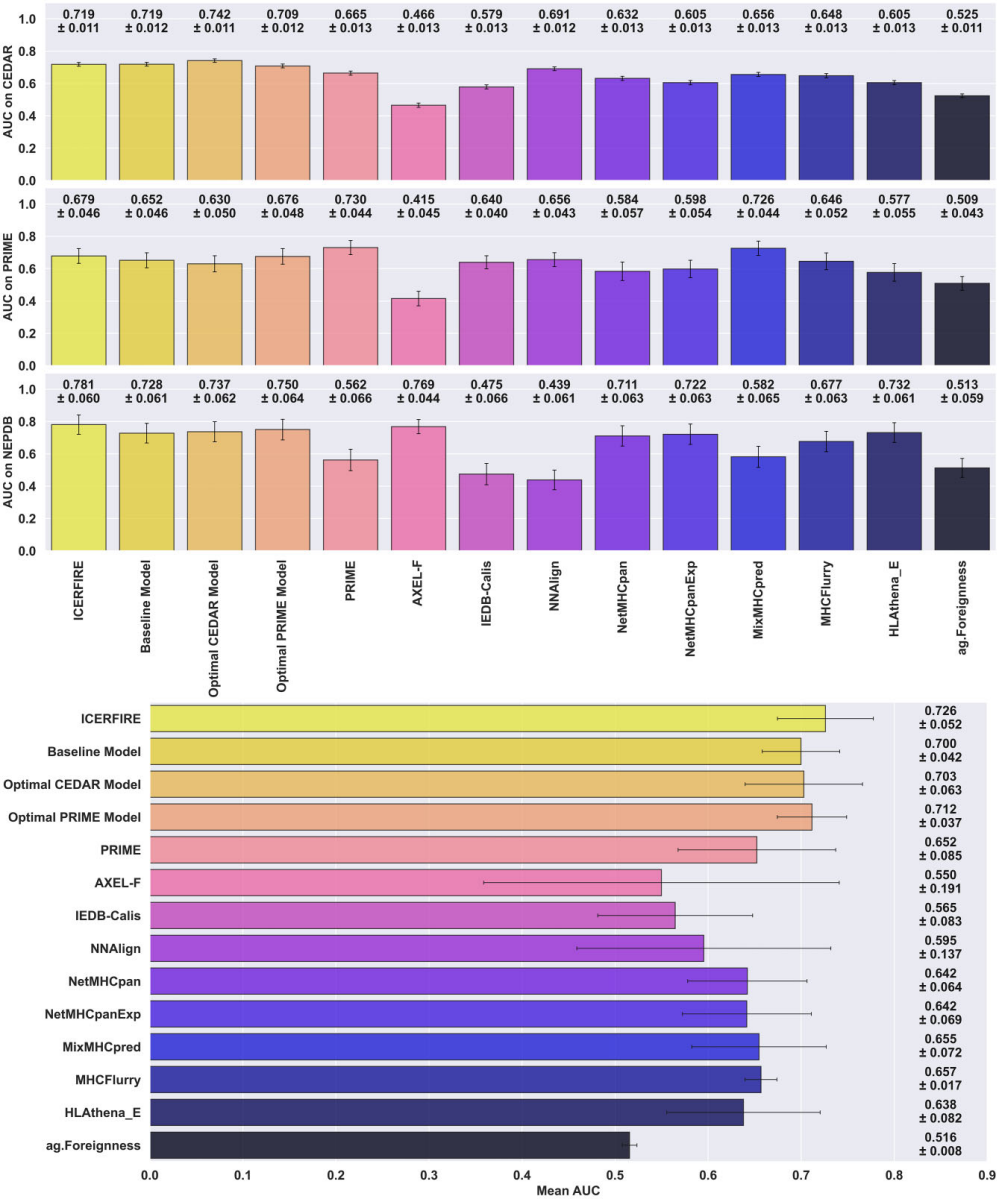
Benchmark of our models and external methods on all three datasets (CEDAR, PRIME, NEPDB). Top panel shows the mean AUCs and standard deviation for 10000 rounds of bootstrapping. Bottom panel reports the mean AUC (*n* = 3) as well as standard deviation across the three datasets. For NetMHCpan, NetMHCpanExp, MixMHCpred, MHCflurry and HLAthena (w/ Expression), one minus the values of the EL ranks, presentation rank and MHC predicted %Rank were used as a score respectively.

The benchmark results (see Figure 7) demonstrate that while the consensus model is not always the top performer, it consistently maintains a high level of performance across all three datasets, yielding the highest average AUC over the three datasets (see Figure 7, bottom panel). We found that while some of the other methods showed strong performance on one dataset, they had significantly lower performance on another dataset. For example, the Optimal CEDAR model yields the best AUC and AUC 0.1 (see Figure 7, figure S6) on the *CEDAR* dataset, and maintains good performance for the *NEPDB* dataset at 0.737 AUC, but fails to generalise to the *PRIME reduced* dataset, with performance dropping to 0.604 AUC. Likewise, the NNAlign model trained on *CEDAR* achieved relatively high performance when evaluated on both *CEDAR* and *PRIME reduced*, with 0.691 and 0.656 mean AUC respectively. This model however failed to carry its predictive power when evaluated on *NEPDB*, with performance dropping to 0.439 AUC, performing worse than random predictions, suggesting overfitting and large differences in motif and characteristics between the neo-epitope datasets.

If we next look at other immunogenicity predictors, a similar dataset specific behaviour was observed when comparing the performances of both PRIME and AXEL-F. For PRIME, while it has the highest AUC on the *PRIME reduced* dataset, which is part of its training data, its AUC and AUC 0.1 values drop to 0.562 and 0.521 respectively on the *NEPDB_expr* dataset. AXEL-F, on the other hand, while it has one of the highest AUC on the *NEPDB_expr* dataset at 0.769, only reaches 0.534 AUC 0.1 whereas our best two models (consensus, optimal PRIME) achieve up to 0.615 AUC 0.1 on the same dataset. More interestingly, when evaluated on the other two datasets, namely *CEDAR* and *PRIME reduced*, AXEL-F’s AUC dramatically drops to 0.466 and 0.415 respectively, performing worse than a model outputting random prediction. The third immunogenicity predictor, IEDB-Calis, did not display such variance across datasets, but rather an overall lower performance in predicting immunogenic neoantigens. This may be caused by the fact that it was developed using only viral peptides, and as such, there is likely a significant difference in the characteristics between viral peptides and neoantigens, limiting the model's predictive power for neoantigen immunogenicity (Figure 7, Figure S6). Lastly, the Foreignness Score (25) alone performs similarly to random predictions, with an average 0.516 AUC across all three evaluation datasets.

Finally, examining the MHC-ligand predictors (NetMHCpan, NetMHCpanExp, MixMHCpred, MHCflurry, HLAthena) (11,12,23,24,32), it is expected that they would have lower predictive power for immunogenicity prediction, as they were not specifically trained for this task, and that the peptides included in the different data set likely, to a very high degree, were selected based on prediction HLA binding. Nevertheless, their performances are still relatively good, outperforming IEDB-Calis and scoring above 0.64 AUC on average and having overall lower variation across datasets than the immunogenicity predictors.

Overall, our consensus model is able to consistently yield high predictive performance over the three datasets, scoring the best overall mean AUC, closely followed by the optimal PRIME model. These results suggest that the other methods are likely overfitting to specific characteristics of their respective training data, rather than generalising well to new data. This is further supported by the mean and standard deviation for AUC and normalised AUC 0.1 values across the three reference datasets (Fig S6).

### Model behaviours and feature importances

**Figure 8.**
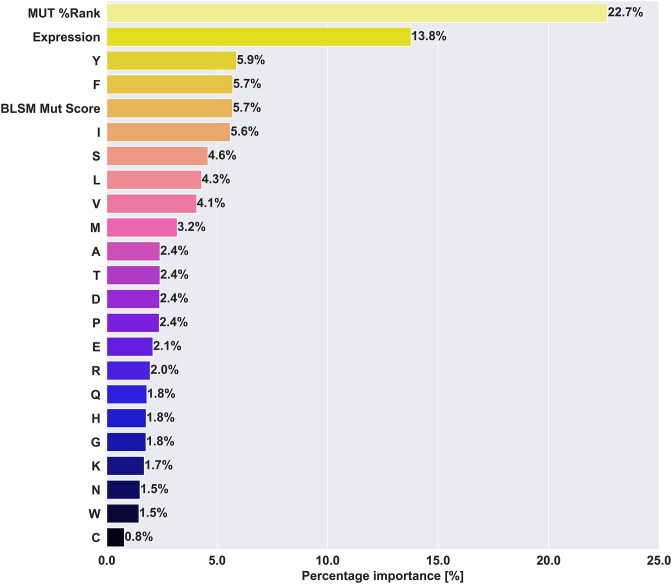
Mean feature importances of the selected consensus model. The feature importances per fold were retrieved and the mean over all the models in the ensemble was calculated for each feature and reported. Feature importances for Random Forest models corresponds to how much a given feature contributes to decreasing the impurity when training and splitting decision trees.

To further illustrate the differences in behaviour between models, we in Figure 8 and Supplementary Figure S5 show the feature importances estimates of each model. In all three models, the most important feature is the percentile rank, with importance values ranging from 14.7 to 22.7%. The optimal PRIME and consensus models behave similarly, giving the BLOSUM mutation score between 4.4 and 5.7% feature importance. (see Figure 8, Supplementary Figure S7). One notable difference between these two models is the usage of scaled agretopicity and self-similarity in the optimal PRIME model, scoring 10.9% and 9.7% of feature importances, while these features are not used in the consensus model. In comparison, the optimal CEDAR model uses a different feature set, with the addition of Boman Index, foreignness score, WT %Rank, while omitting the ICORE self-similarity score. Together, these additional features take up 22.2% of the feature importances, while the BLOSUM mutation score's importance drops to 2.5%, from around 4.7% for the other models. This shift, along with the positional weighting scheme may be an indication of overfitting, with the model's inability to generalise to the PRIME dataset. When it comes to the feature importance of Tryptophan (W), despite previous studies observing an enrichment of this amino acid (30,42) in immunogenic peptides, it consistently ranks at the bottom three of the feature importances in all three models, with less than 1.5% feature importance on average.

Schmidt et al. included both random human self-peptides and viral data in their training dataset (30). To uncover whether either data type could influence the feature importance of tryptophan, we complemented our training dataset with progressively increasing amounts of viral or self-peptides. The results summarised in Supplementary Figure 8 revealed a notable change in the feature importance of Tryptophan, showing an increasing trend only with the addition of viral data. In addition, we investigated the enrichment of Tryptophan (W) in immunogenic peptides within both the viral and neo-epitope datasets (refer to Supplementary Table 4). We observed a significant enrichment of tryptophan in positive peptides in the viral dataset, measuring 0.0187 and 0.0069 for immunogenic and non-immunogenic peptides, respectively (*P* = 2.35e-37, one-sided Welch's *t*-test). In contrast, the mean proportion of W in the neo-epitope dataset did not show a significant enrichment, measuring 0.0176 and 0.0161 for immunogenic and non-immunogenic neo-epitopes, respectively (*P* = 0.236, one-sided Welch's *t*-test). Our results demonstrate that as the proportion of viral data in the training set increased, the tryptophan feature importance increased accordingly, up to 8% for the case of 81.8% of viral data in the training dataset, while neo-epitope AUC dropped down to around 60%. This finding suggests that the previously observed enrichment of Tryptophan in immunogenic peptides might be attributed to the addition of viral data rather than a specific enrichment in immunogenic neo-epitopes or self-peptides. Furthermore, it is worth noting that the performance of the models in predicting immunogenicity in neo-epitopes decreased as we added more viral or self-peptide data. These findings suggest that the addition of mixed data types does not necessarily improve the model's ability to predict immunogenic neo-epitopes. Instead, the previously reported performance by other studies might be attributed to the model's improved capability to identify viral immunogenic peptides or non-immunogenic self-peptides. These findings highlight the complexity of feature importance and emphasise the importance of carefully considering the composition of the training dataset, as it can significantly impact both feature importance and prediction performance.

### Web server interface and usage

The trained model is accessible through a web server hosted at https://services.healthtech.dtu.dk/services/ICERFIRE-1.0/. Input data must be provided in a comma-separated format, with information about the mutant, wild-type, and HLA allele. The user may also provide their own antigen expression values (in TPMs) as an additional column. The web server employs NetMHCpan-4.1 (23) to initially predict HLA-binding affinities as %Rank and ICOREs. Subsequently, the aligned wild-type ICOREs are identified to generate the feature set that serves as input for the model. Furthermore, users have the option to select between a model trained with expression (by default) or without expression. In cases where expression values are included, the server automatically queries a reference database (TCGA-pancan (17)) to retrieve wild-type expression values using PepX. Any missing values are substituted with the median TPM value from the reference database.

The program's output provides a comprehensive view of immunogenicity predictions, shedding light on the potential immune response tied to the input data. Key components of the output include NetMHCpan rank predictions, quantifying the likelihood of HLA antigen presentation in terms of percentile rank. It extends further to include the predicted ICOREs for mutants, predicted aligned ICOREs for their wild-type counterparts, and features like gene TPM values, self-similarity scores, and BLOSUM mutation scores. The immunogenicity predictions are complemented by a percentile rank score, spanning from 0 to 100 (in percentages), where a rank of 0 corresponds to a highly immunogenic peptide. The percentile ranks are obtained by scoring the model against ∼110 000 randomly selected human proteome peptides, each with random mutations (for details refer to Materials and methods).

## Discussion

The identification of suitable antigen targets is vital for the development of personalised cancer immunotherapies, such as cancer vaccines. Immunogenicity prediction plays a critical role in this process, as it enables the characterisation of relevant epitopes (7,10,49–51). In this study, we developed ICERFIRE, a model that predicts the immunogenicity of neo-epitopes using an ensemble of Random Forest models trained on curated neo-epitope data from CEDAR (33). We explored an array of previously studied and novel features to develop a model able to better generalise to multiple datasets. The final model is an ensemble of 90 Random Forest models, developed by nested cross-validation, with each fold constructed using data redundancy reduction methods to prevent data leakage and reduce overfitting.

The study aimed to identify optimal models and peptide features for predicting immunogenic neo-epitopes using different datasets. Extensive searches were conducted to find combinations of features and weighting schemes that yielded the best-performing models. In terms of the peptide sequence, the optimal input to the model was found to be the amino acid composition of the predicted ICORE of the tumour associated peptide, i.e. the nested submer of the reported peptide with optimal predicted HLA antigen presentation potential, combined with its predicted likelihood of antigen presentation (%Rank). This observation was robustly found across all investigated data sets.

However, from our analysis of the optimal models, it became evident that models were often overfitted to specificities of each given datasets. By way of example, the optimal CEDAR model incorporated the Inverted (IC) positional weighting, upweighted anchor positions, and additional features such as the Boman Index, BLOSUM mutation score, WT %Rank, foreignness score, and antigen expression. It outperformed the baseline ICORE model in cross-validation. However, this model demonstrated a significant performance drop when evaluated on the alternate PRIME dataset, suggesting poor power of generalisation. Furthermore, despite the inclusion of the foreignness score as a feature in this model, it only accounts for 1.7% of feature importances (see Figure S7), ranking at the bottom five least important features along with the amino acids cysteine, histidine, asparagine and tryptophan.

In comparison, the optimal PRIME model used masking as positional weighting, with self-similarity, BLOSUM mutation score, scaled agretopicity, and antigen expression as additional features, also outperforming the baseline ICORE model. In contrast, the ‘consensus’ ICERFIRE model, which balanced cross-validation AUC on CEDAR and test AUC on PRIME, showed comparable performance on both datasets. Together, our results suggest that the large differences in optimal features as well as positional weighting methods highlight the challenges of generalisation and the potential limitations of dataset-specific models in predicting immunogenic neo-epitopes. The performance of ICERFIRE was found to rely on positional weighting to mask out anchor positions. Our analysis further identified features that relate neo-epitopes to their wild-type counterparts, such as the BLOSUM mutation score and peptide abundance (in the form of gene expression) to improve prediction accuracy.

The main obstacles in developing accurate neo-epitope immunogenicity predictors are data heterogeneity and quality and quantity of data. The first point to address is the bias in HLA restriction. In our training dataset, out of 65 alleles present in the data, peptides restricted to the top 10 most common alleles make up 84.47% of the dataset, with peptides restricted to HLA-A*02:01 constituting 38.35% of all peptides. This overrepresentation of certain alleles in the training data can affect the model's performance when evaluated on peptides restricted to lesser-known alleles. Secondly, the definition of immunogenicity from a data perspective is not always consistent. Akin to IEDB, data from CEDAR is curated from different studies with a myriad of T cell assay types, both *in vitro* and *in vivo*. Three main categories can be used to filter assays (3D structure, biological activity, binding), of which many sub-categories exist, for instance cytokine release (IL-2, IFN-γ) or degranulation detection for biological activity. Immunogenicity can thus be defined as a positive observation in any of the aforementioned assays. However, a peptide that was found to be immunogenic *in vitro*, for example, through an ELISPOT detection of cytokine release might not necessarily elicit an immune reaction *in vivo*. Thirdly, a single peptide might have multiple entries, with both positive and negative reactions to the same or different assays, where some assays have different levels of quantitative labels (negative, positive low, positive high). To train our models, all the labels for a given Peptide-HLA pair were collapsed into a single class label, where a data point is deemed immunogenic if there is a single positive entry, and negative only if all the entries are negative. While this approach is intuitive and simple, it has important potential negative implications. For instance, a peptide with a single negative data entry will be labelled as non-immunogenic despite the fact that such a claim clearly would require the peptide being tested in multiple assays and individuals to deal with issues such a immunodominance and immune-stochasticity (52,53). Even though availability of more data to some extent could alleviate this problem, the unique and personalised nature of cancer genomes makes it an inherent, natural and unsolvable property of neo-epitopes, limiting the degree of annotation accuracy for individual peptides, placing a cap on the performance of the developed method for prediction of neo-epitope immunogenicity. Finally, a fundamental issue lies in the current curation of the data. We observed that several peptides annotated as neo-epitopes in the database did not meet the true definition of neo-epitopes. Instead, these peptides were peptide analogs where a specific anchor amino acid was substituted with an alternative amino acid, resulting in altered binding properties. For instance, the peptide SLLMWITQV was identified as a neo-epitope, replacing the amino acid C at position 9 with V to enhance binding to HLA-A*02:01. However, this alteration is not associated with a tumour mutation, but is rather a mutation artificially introduced to increase MHC binding. A similar situation arises with the synthetic peptide ELAGIGILTV, which is an anchor-optimised variant of the WT peptide EAAGIGILTV, wherein the amino acid A at position 2 is replaced with L to enhance binding to HLA-A*02:01. To address this issue, an improved curation and filtering process identifying true neo-epitopes derived from cancer mutations is critical in facilitating the development of immunogenicity prediction models for neo-epitopes.

Other studies previously observed an enrichment of tryptophan in immunogenic peptides (30,42). However, when looking at the feature importances of our selected models, frequency of tryptophan consistently appears among the least important features. This is in line with observations from earlier work related to cancer neo-epitope (52). We hypothesise that this discrepancy could stem from the type of training data included. While our models are restricted to neo-epitopes only, both PRIME (30) and IEDB-Calis (42) were developed by adding or using only viral peptides. In the context of immunogenicity prediction for neo-epitopes, we tested whether the inclusion of viral data had an effect on both the feature importance of tryptophan and the performance of our models when evaluated on neo-epitopes. We trained the base model and added increasing amounts of viral data from IEDB in the training set, reporting the tryptophan importance and cross-validation AUC on neo-epitopes only versus the amount of viral data added (see Figure S8). This suggests that the enrichment in tryptophan noted by Schmidt *et al.* and Calis *et al.* could be attributed to the increased frequency of the amino acid in viral data and could explain the poor performance of the IEDB-Calis model when benchmarked on neo-epitope datasets as shown in the results section.

In summary, in this study, we have investigated the prediction of immunogenic neo-epitopes using a large set of features and input methods. Our findings demonstrated the importance of feature selection and weighting schemes in achieving optimal predictive performance, while highlighting the potential bias from using certain features depending on the dataset. While both trained on the CEDAR dataset, the optimal CEDAR and PRIME models showed promising results on their respective test datasets. However, the former exhibited poor generalisation capabilities compared to the latter. An ‘consensus’ model, designed to balance performance across datasets, emerged as a viable alternative, suggesting the potential for a more generalised approach, although this remains unsolved. In our benchmark, we further highlighted overfitting issues in several models, both in our and the external methods, highlighting the need for cautious model selection and training on unbiased data. Future research should explore ways to improve dataset curation. Data gathered from various studies and databases could be mixed to generate a larger training dataset. However, independent validation datasets are equally important, emphasising the need for a larger amount of robust, unbiased data. Finally, the exploration of additional features to improve the predictive accuracy of immunogenic neo-epitope models should consider potential biases to prevent overfitting. Overall, this study contributes to the understanding of predictive modelling in immunoinformatics and provides valuable insights for the development of more accurate and generalised models for immunogenic neo-epitope prediction.

## Supplementary Material

zcae002_Supplemental_Files

## Data Availability

The web server for the model described in this work is available at https://services.healthtech.dtu.dk/services/ICERFIRE-1.0/. The datasets used to train, evaluate, as well as the benchmarks prediction scores are available in the supplementary data section at NAR online.
