# Supplementary material for: A large-scale study of peptide features defining immunogenicity of cancer neo-epitopes
Source: NAR Cancer. 2024 Jan 29;6(1):zcae002. doi: 10.1093/narcan/zcae002 (PMC10823584; doi:10.1093/narcan/zcae002)

SUPPLEMENTARY DATA

For the supplementary tables, please refer to the data uploaded to the NARC submission page. The tables are named Table_SN_XYZ, where N is the number of the table as referred to in the main text, and XYZ its descriptive filename.

Figure S1 Input comparison between full peptide and ICORE. Receiving Operator Curves were generated by using the 1 minus the predicted %Rank, of either the full peptide or the optimal ICORE as prediction score for immunogenicity.

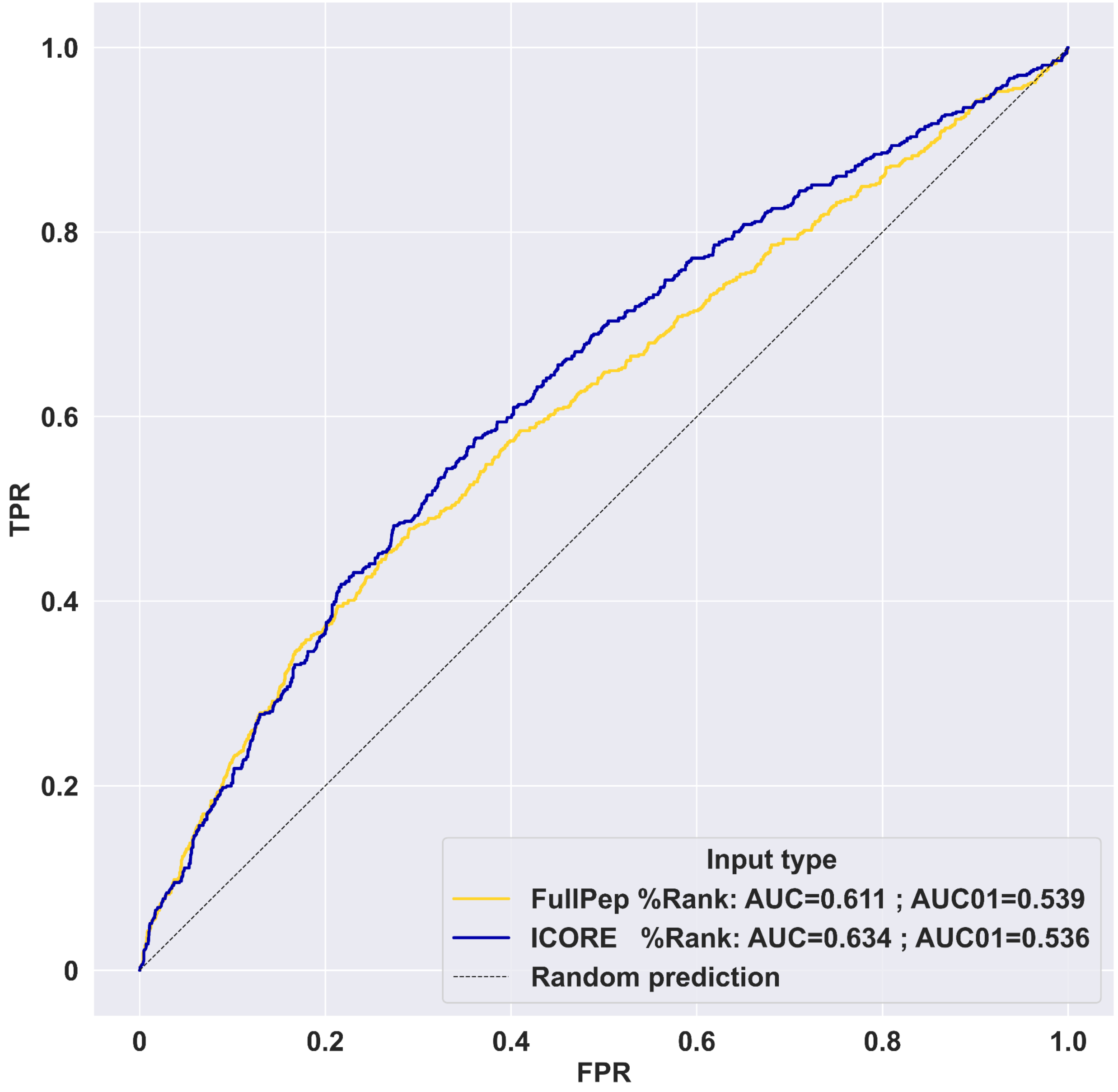

Figure S2: All Input and weights comparison. For models trained and evaluated on CEDAR using as input either the full peptide (top panel), the ICORE (middle panel) or the expanded binding CORE allowing for insertions (bottom panel)

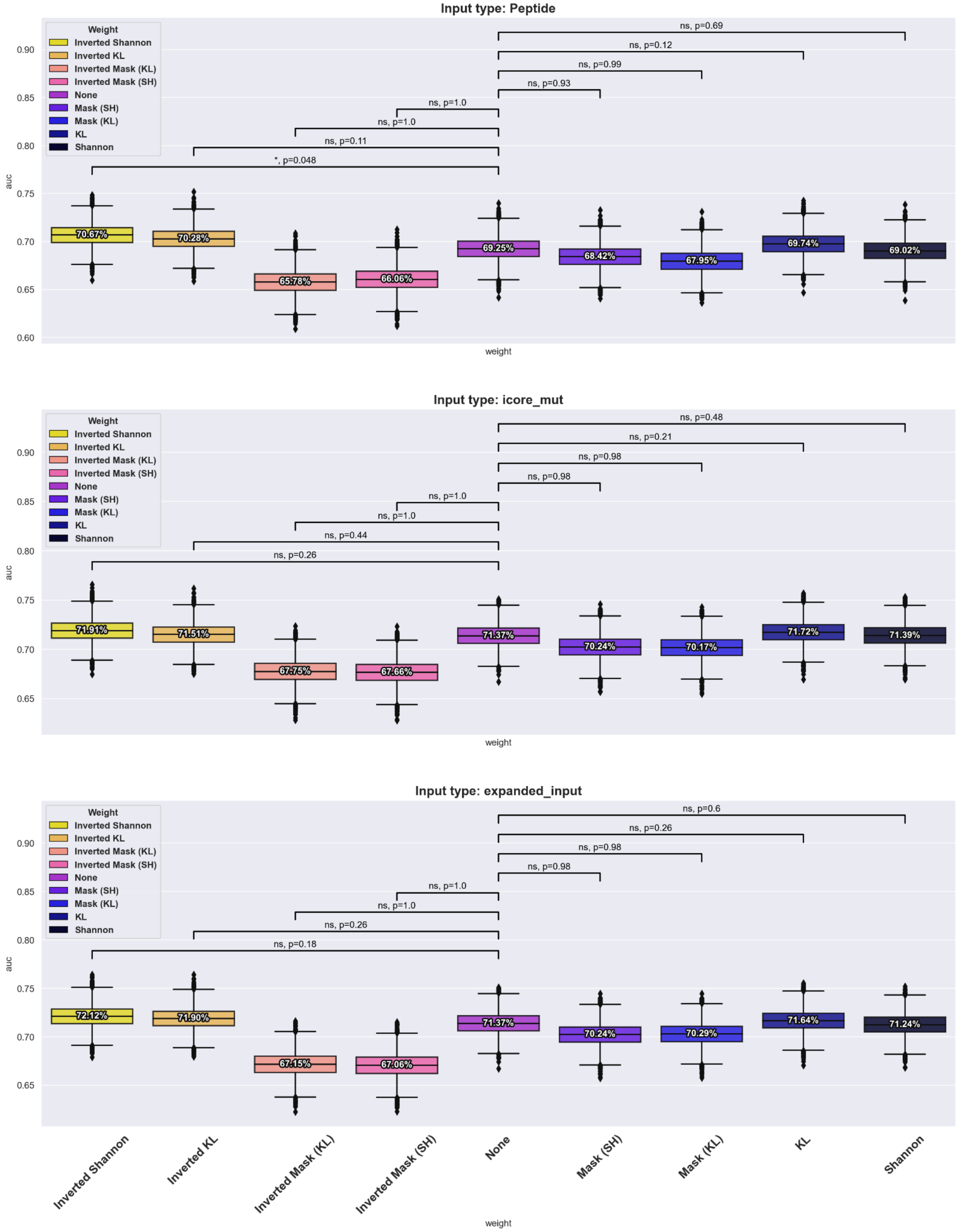

Figure S3: Mutant, WT EL %Rank grouped by mutation type and immunogenicity. Top panel summarises Log of predicted %Ranks grouped by mutation type and label. Middle panel reports Mut %Rank on the X and WT %Rank on the Y axis. Bottom panel summarises the distribution of self-similarity with respect to anchor vs non-anchor mutation groups and immunogenicity.

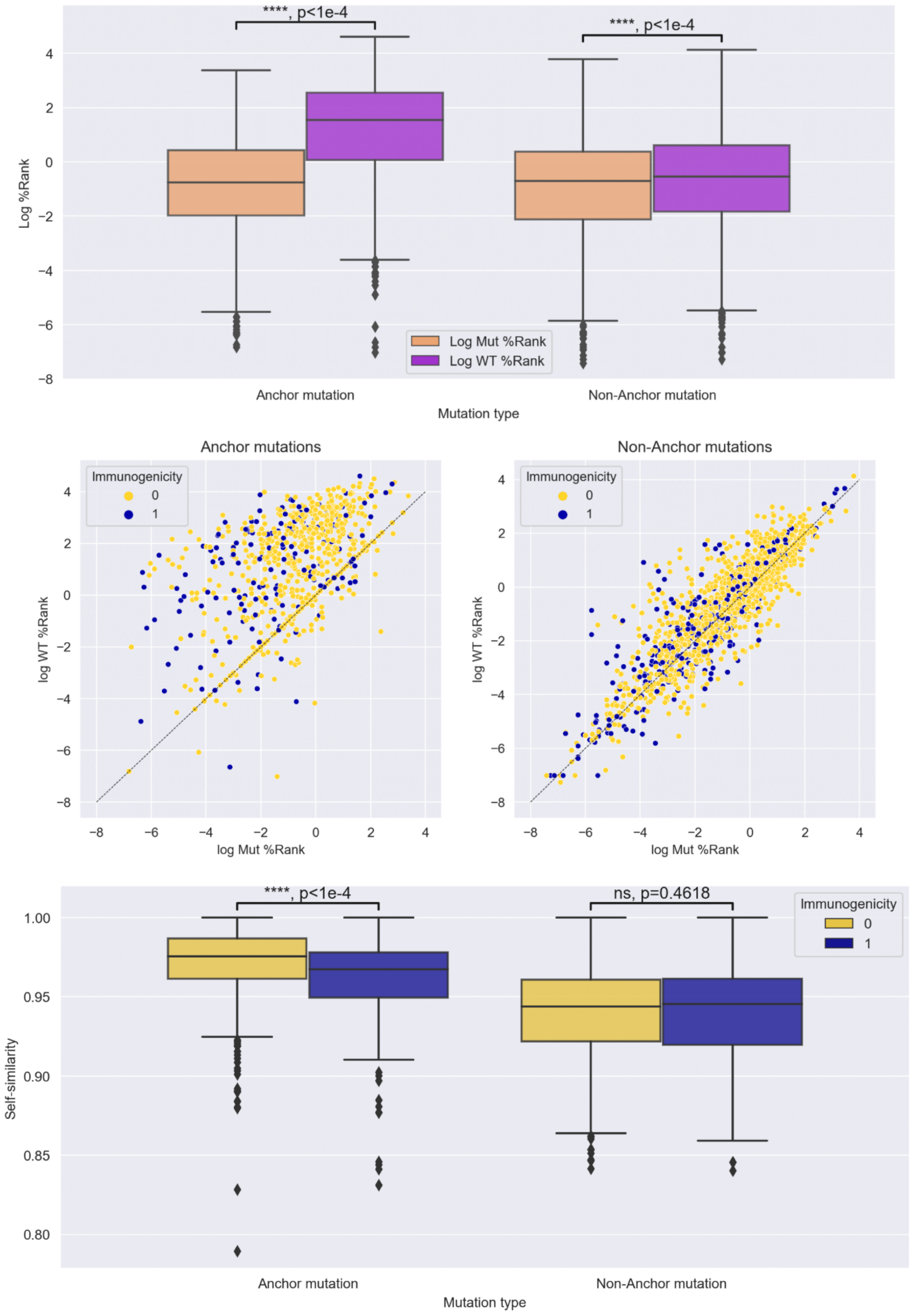

Figure S4: Distribution of selected features per label. With AUC values computed by using each feature as a score.

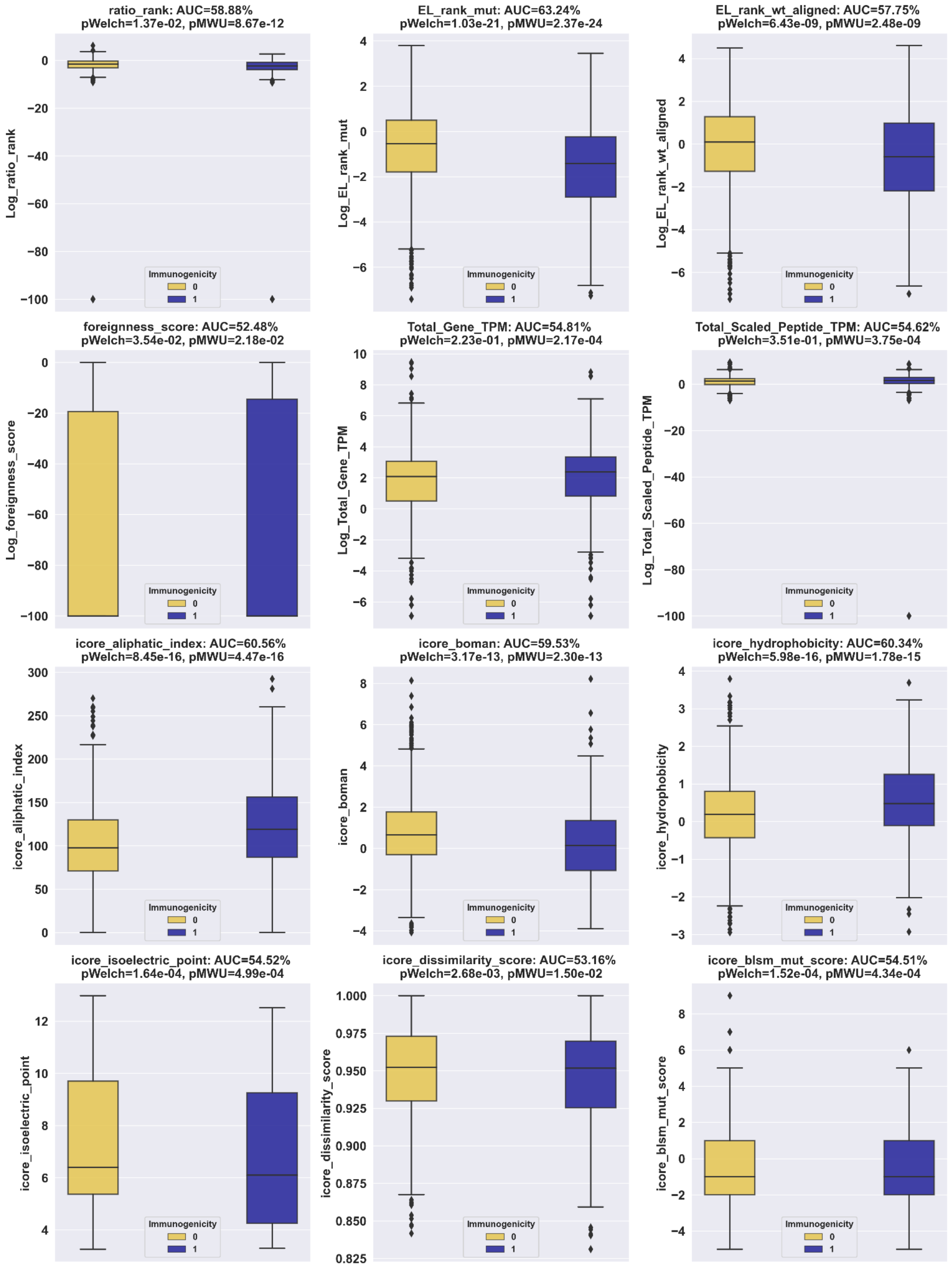

Figure S5: Correlation matrix of features for the CEDAR dataset

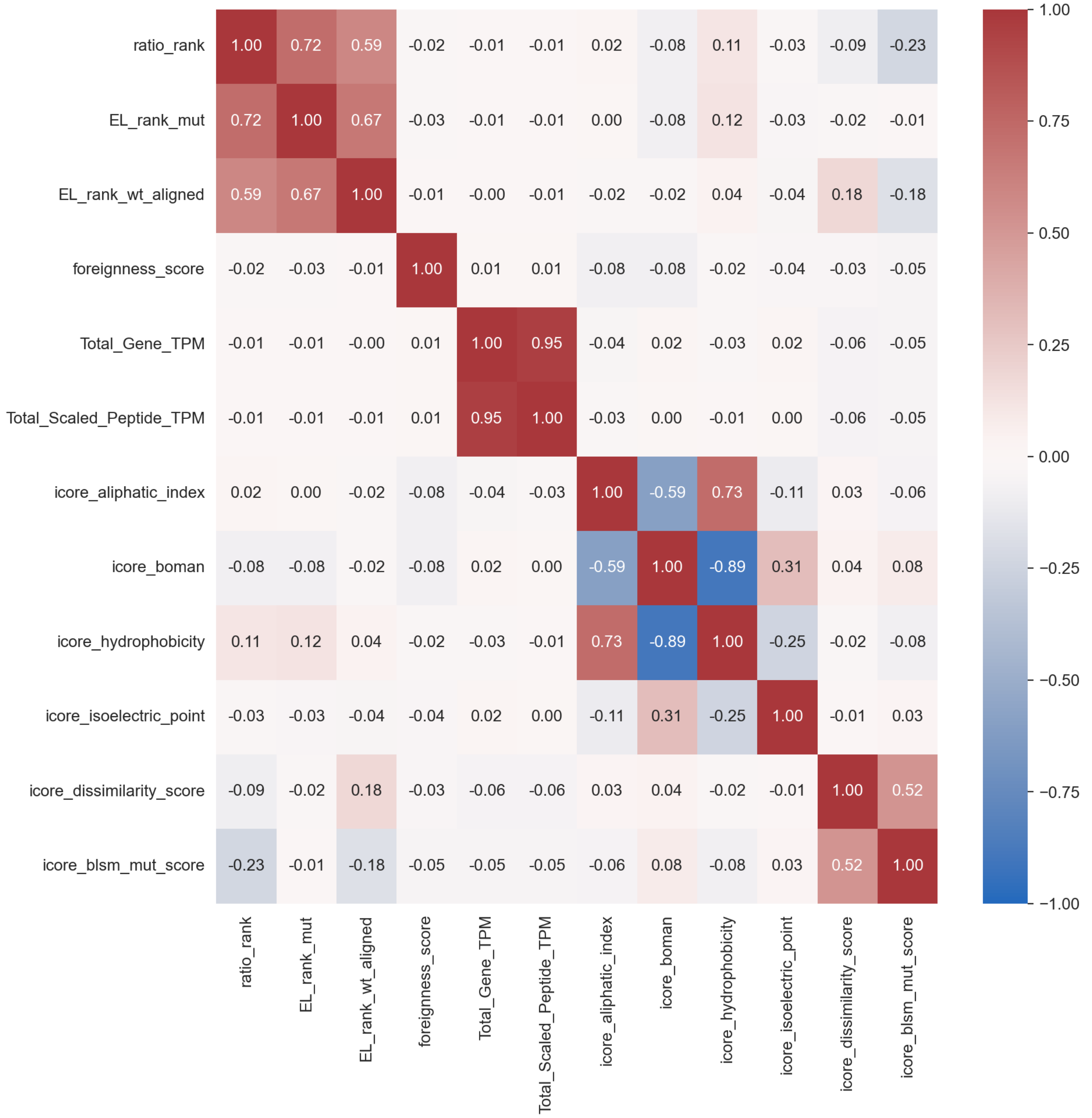

Figure S6: AUC 01 for the benchmark of our models and external methods on all three datasets (CEDAR, PRIME, NEPDB). Top panel shows the mean AUC 01 and standard deviation for 10000 rounds of bootstrapping. Bottom panel reports the mean AUC 01 (n=3) as well as standard deviation across the three datasets. For NetMHCpan, NetMHCpanExp, MHCflurry, and HLAthena (w/ Expression), one minus the values of the EL rank, presentation rank, and MHC predicted %Rank were used as a score respectively.

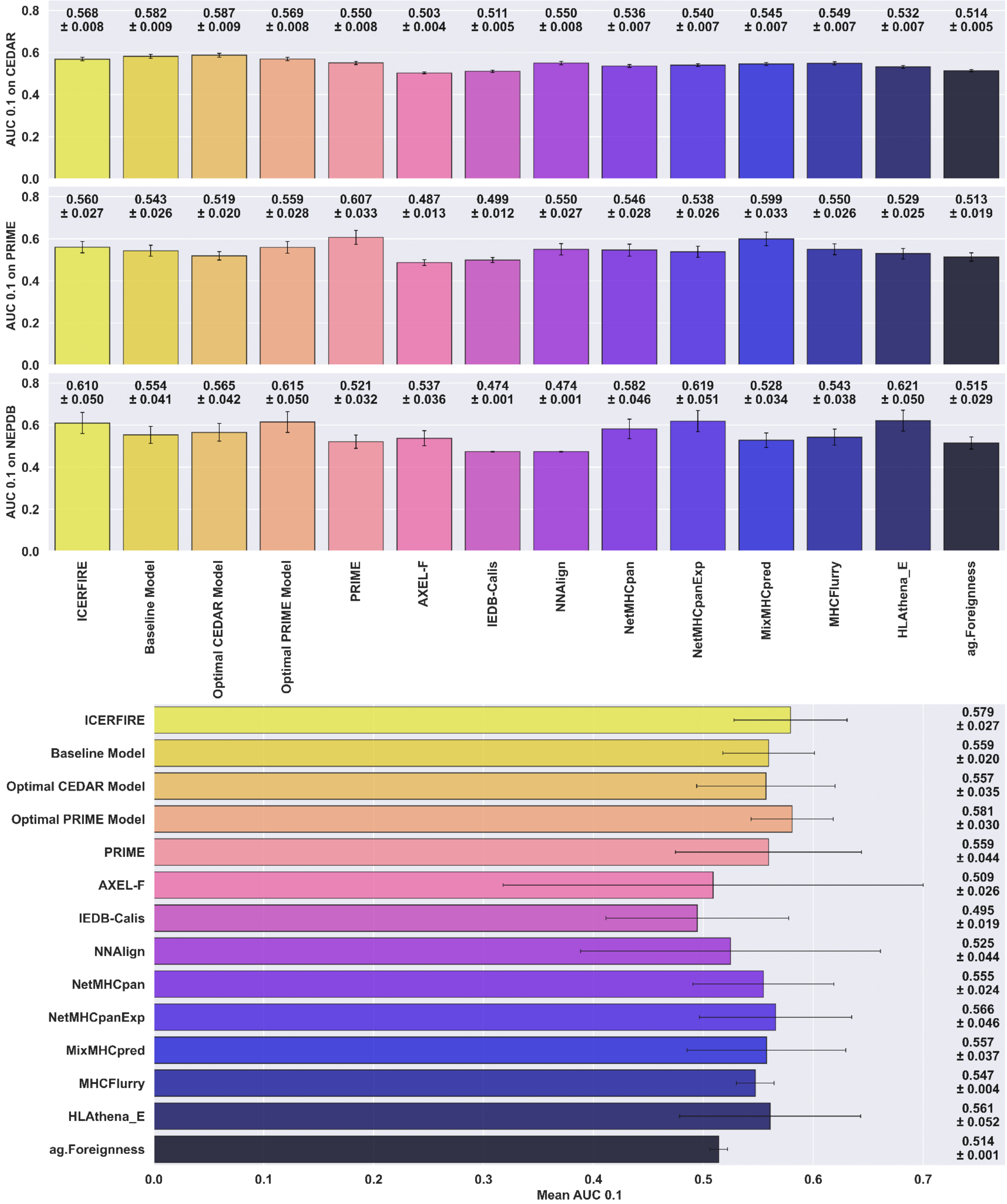

Figure S7: Mean feature importances of the Optimal CEDAR and Optimal PRIME model. For each model, the feature importances per fold were retrieved and the mean over all the models in the ensemble was calculated for each feature and reported. Feature importances for Random Forest models corresponds to how much a given feature contributes to decreasing the impurity when fitting and splitting decision trees.

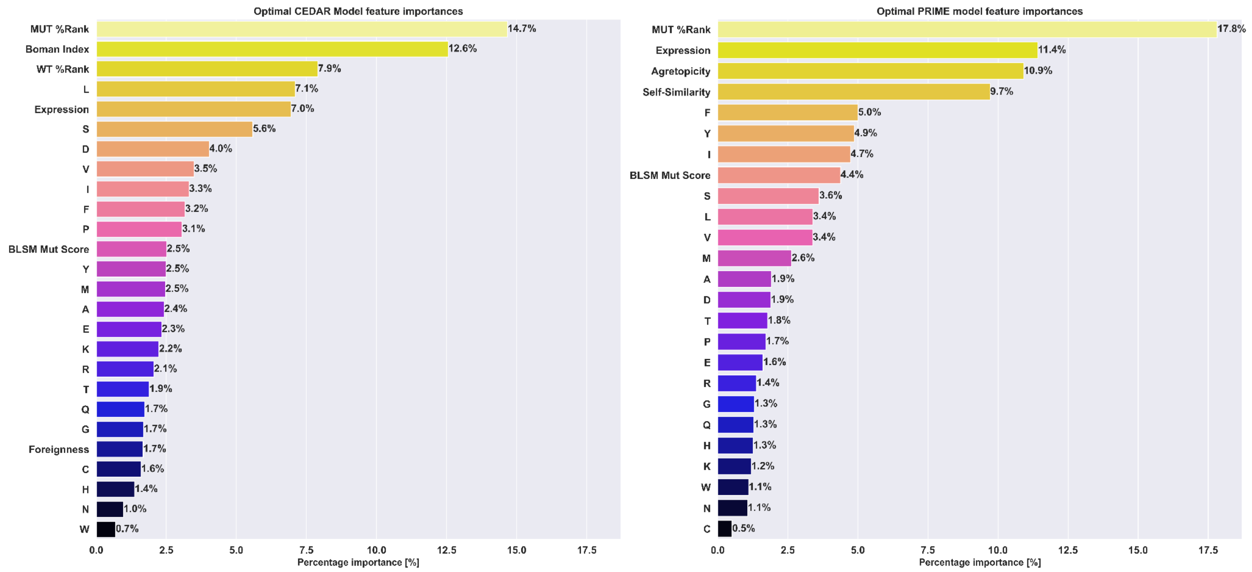

Figure S8: Tryptophan feature importance and AUC of models evaluated on neo-epitopes as a function of the proportion of viral data or human peptides data in the training set. The top two panels indicate feature importance of W and performance measured in ROC AUC on neo-epitopes for models trained on neo-epitopes + viral data. Bottom two panels show feature importance and performance for models trained on neo-epitopes + human peptide data. Tryptophan feature importance increases with the proportion of viral data but not the proportion of self-peptide data present in the training dataset. AUC for neo-epitope prediction generally decreases as the proportion of neo-epitope data within the training dataset decreases.

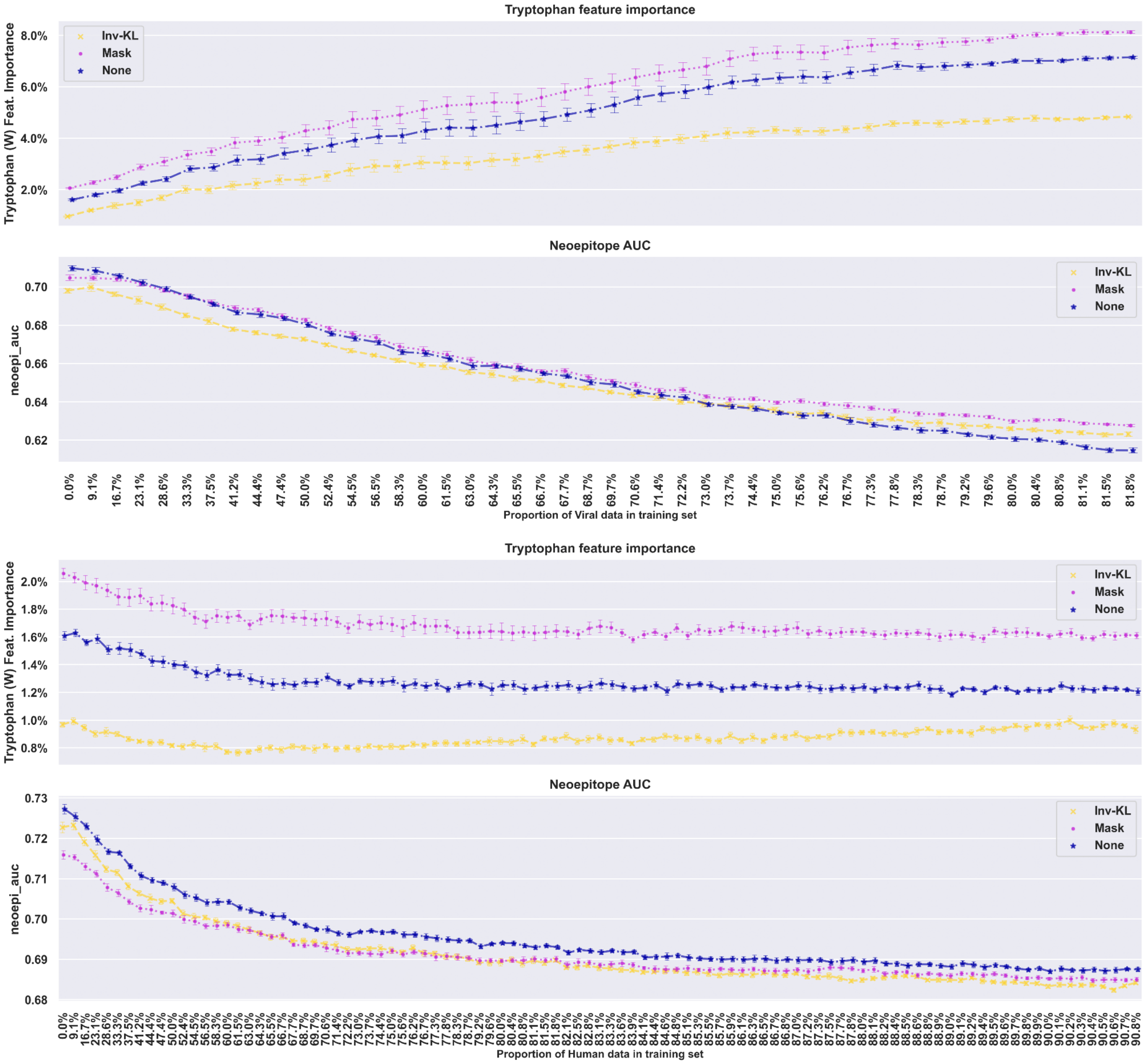

Supplement: zcae002_Supplemental_Files [file zcae002_supplemental_files.zip › ICERFIRE_NAR_Supplementary.pdf]
